# Discovery of *Bacteroides uniformis* F18-22 as a Safe and Novel Probiotic Bacterium for the Treatment of Ulcerative Colitis from the Healthy Human Colon

**DOI:** 10.3390/ijms241914669

**Published:** 2023-09-28

**Authors:** Wei Dai, Jiaxue Zhang, Lu Chen, Junhong Yu, Junyi Zhang, Hua Yin, Qingsen Shang, Guangli Yu

**Affiliations:** 1 State Key Laboratory of Biological Fermentation Engineering of Beer, Qingdao 266199, China; daiwei3266@stu.ouc.edu.cn (W.D.); chenlu@tsingtao.com.cn (L.C.); yujh@tsingtao.com.cn (J.Y.); 2 Key Laboratory of Marine Drugs of Ministry of Education, Shandong Provincial Key Laboratory of Glycoscience and Glycotechnology, School of Medicine and Pharmacy, Ocean University of China, Qingdao 266003, China; zhangjiaxue@stu.ouc.edu.cn (J.Z.); glyu@ouc.edu.cn (G.Y.); 3 Qingdao Marine Biomedical Research Institute, Qingdao 266071, China; zhangjunyi@ouc.edu.cn; 4 Laboratory for Marine Drugs and Bioproducts, Qingdao National Laboratory for Marine Science and Technology, Qingdao 266003, China

**Keywords:** *Bacteroides uniformis*, gut microbiota, ulcerative colitis, dextran sulfate sodium, probiotic, alginate, inflammatory bowel disease, *Eubacterium siraeum*, fermentation, short-chain fatty acids

## Abstract

Previous studies have demonstrated that the intestinal abundance of *Bacteroides uniformis* is significantly higher in healthy controls than that in patients with ulcerative colitis (UC). However, what effect *B. uniformis* has on the development of UC has not been characterized. Here, we show for the first time that *B. uniformis* F18-22, an alginate-fermenting bacterium isolated from the healthy human colon, protects against dextran-sulfate-sodium (DSS)-induced UC in mice. Specifically, oral intake of *B. uniformis* F18-22 alleviated colon contraction, improved intestinal bleeding and attenuated mucosal damage in diseased mice. Additionally, *B. uniformis* F18-22 improved gut dysbiosis in UC mice by increasing the abundance of anti-inflammatory acetate-producing bacterium *Eubacterium siraeum* and decreasing the amount of pro-inflammatory pathogenetic bacteria *Escherichia-Shigella* spp. Moreover, *B. uniformis* F18-22 was well-tolerated in mice and showed no oral toxicity after repeated daily administration for 28 consecutive days. Taken together, our study illustrates that *B. uniformis* F18-22 is a safe and novel probiotic bacterium for the treatment of UC from the healthy human colon.

## 1. Introduction

Ulcerative colitis (UC) is one type of inflammatory bowel disease (IBD) that is characterized by bacterial dysbiosis in the gut [1,2,3]. Gut dysbiosis contributes significantly to the pathogenesis of UC and new therapeutic techniques, including fecal microbiota transplantation (FMT), and live biotherapeutic products (LBPs) have been developed for the treatment of IBD by targeting the dysbiotic microbiome [4,5,6]. Recently, accumulating evidence has indicated that the intestinal abundance of *Bacteroides uniformis* is significantly higher in healthy controls than that in patients with UC [7,8], suggesting a potential role of this bacterium in the pathogenesis of colonic diseases. However, up to now, what effect *B. uniformis* has on the development of UC has not been characterized.

Previous studies have demonstrated that specific strains of *B. uniformis* are next-generation probiotic bacteria that can be used to improve exercise performance both in mice and humans [9]. Besides, others found that oral administration of *B. uniformis* could ameliorate the metabolic and immunological dysfunction in mice with high-fat-diet-induced obesity [10]. Additionally, oral intake of *B. uniformis* was also observed to amplify the metabolic and immune benefits of dietary fiber by targeting gut dysbiosis in obese mice [11]. Collectively, these studies indicate that *B. uniformis* might be used as a novel probiotic bacterium for the treatment of dysbiosis-associated colonic diseases.

Alginate is a fermentable dietary fiber that could be utilized by specific microbes in the human gut [12,13]. Our previous results indicated that the alginate-fermenting bacterium, *Bacteroides xylanisolvens* AY11-1, could mediate the beneficial effects of alginate on the colon and oral administration of *B. xylanisolvens* AY11-1 protects against dextran-sulfate-sodium (DSS)-induced UC in mice [12]. Similar to that of alginate, *B. xylanisolvens* AY11-1 improved gut dysbiosis and promoted the growth of probiotic bacteria in diseased mice [12]. This study highlights the importance of exploring alginate-fermenting bacteria for the discovery of potential anti-colitis probiotics from the human colon. 

In the present study, combined with the observation that the intestinal abundance of *B. uniformis* is significantly higher in healthy controls than that in IBD patients, we hypothesized that the alginate-fermenting bacterium *B. uniformis* F18-22 might also be protective against the development of UC [12]. We therefore tested this hypothesis in mice and interestingly, we found that *B. uniformis* F18-22 could be used as a safe and novel probiotic bacterium for the treatment of UC from the healthy human colon. 

Our study consists three parts of experiments. In the first part, we sequenced the whole genome of *B. uniformis* F18-22 and explored its metabolic potential. In the second part, we investigated the anti-colitis effect of *B. uniformis* F18-22 and its potential therapeutic mechanisms using a mouse model of DSS-induced colitis. In the third part, we further examined the safety profiles of *B. uniformis* F18-22 in vivo. All the three parts of the experiments were designed to provide the first evidence for the potential utilization of *B. uniformis* F18-22 as a next-generation probiotic. 

## 2. Results

### 2.1. Genomic Analysis of the Alginate-Fermenting Bacterium, B. uniformis F18-22

**Figure 1:**
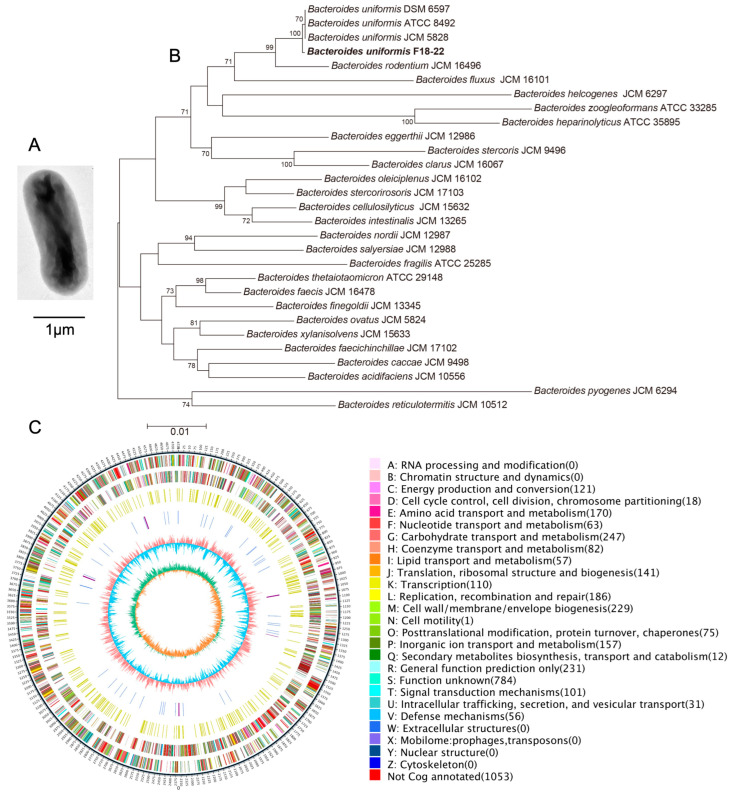
Genomic analysis of *B. uniformis* F18-22. TEM analysis of the bacterial cell morphology of *B. uniformis* F18-22 (**A**). Phylogenetic tree analysis of *B. uniformis* F18-22 based on 16S gene sequences (**B**). COGs function analysis of *B. uniformis* F18-22 (**C**).

Gut microbiota plays a critical role in the metabolism of nutrients and as a dietary fiber, alginate could be fermented by the human gut microbiota [12,13]. *B. uniformis* F18-22 is an alginate-fermenting bacterium that has previously been isolated from the healthy human colon [12]. In the present research, we aim to extend our previous study by exploring the metabolic characteristics and the anti-colitis effect of *B. uniformis* F18-22. A transmission electron microscope (TEM) was first employed to investigate the cell morphology of *B. uniformis* F18-22. TEM analysis indicated that *B. uniformis* F18-22 is a rod-shaped bacterium with a cell length of about 2.5 μm (Figure 1A). Phylogenetic tree analysis suggested that *B. uniformis* F18-22 is closely related to other strains in the same species, including the type strain *B. uniformis* ATCC 8492 (Figure 1B). 

We next sequenced the whole genome of *B. uniformis* F18-22 to explore the metabolic potential of this bacterium. The length and the GC content of the genome of *B. uniformis* F18-22 was identified to be 4,725,126 bp and 46.59%, respectively (Figure 1C). Clusters of Orthologous Group (COG) function analysis indicated that *B. uniformis* F18-22 has a good capability for carbohydrate transport and metabolism, and a total of 247 identified COGs were annotated in this category (Figure 1C). This indicates that *B. uniformis* F18-22 might be able to degrade and ferment a wide range of different dietary polysaccharides in the human gut. This could possibly give *B. uniformis* F18-22 a competitive advantage to survive in the colon when the carbon source is changed between different diets.

**Figure 2:**
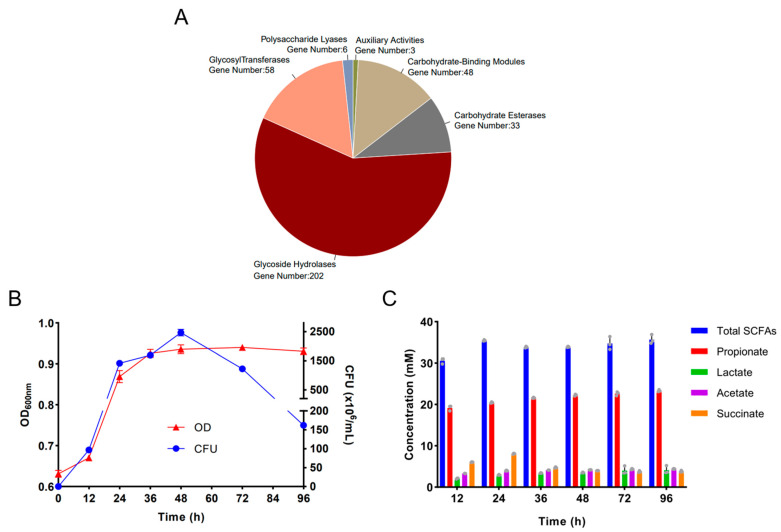
CAZymes analysis of *B. uniformis* F18-22 and in vitro fermentation of alginate by *B. uniformis* F18-22. Genomic CAZymes analysis of *B. uniformis* F18-22 (**A**). CFUs analysis and growth curve analysis of *B. uniformis* F18-22 during fermentation of alginate (**B**). SCFAs analysis of *B. uniformis* F18-22 produced during fermentation of alginate (**C**). The gray dots in the panel C represent the three replicates in the experiments.

Carbohydrate-active enzymes (CAZymes), including glycoside hydrolases (GHs), glycosyltransferases (GTs), polysaccharide lyases (PLs), carbohydrate esterases (CEs), carbohydrate-binding modules (CBMs), and auxiliary activities (AAs), are the most important enzymes for the metabolism of complex carbohydrates in the human diet. We next investigated the CAZymes in *B. uniformis* F18-22 and interestingly, a total of 350 genes were identified as responsible for the expression of a different class of CAZymes (Figure 2A). Remarkably, over one half of these genes were identified as responsible for the expression of glycoside hydrolases (GHs) (Figure 2A). In line with previous results [12], further study confirmed that *B. uniformis* F18-22 could ferment and utilize alginate for its own growth (Figure 2B). *B. uniformis* F18-22 can grow up to 2.5 × 10^9^ CFUs/mL in about 48 h in the medium containing alginate as a major carbon source (Figure 2B). Moreover, fermentation of alginate by *B. uniformis* F18-22 produced significant amounts of beneficial short-chain fatty acids (SCFAs), including propionate, lactate, acetate, and succinate (Figure 2C). The majority of the produced SCFAs was propionate, and the minority of the produced SCFAs was lactate, acetate, and succinate (Figure 2C). 

### 2.2. B. uniformis F18-22 Attenuated DSS-Induced UC in Mice

**Figure 3:**
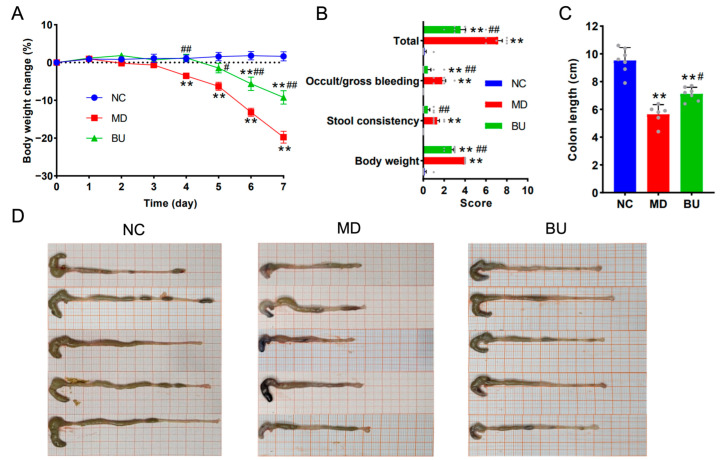
Oral intake of *B. uniformis* F18-22 attenuated DSS-induced UC in mice. Body weight change of the mice (**A**). Symptom score analysis of the disease (**B**). Colon length analysis of the mice (**C**). Representative morphology of the colon (**D**). Each gray dot in the panel B and C represents one mouse in the experiments. The NC group has 7 mice. The MD group has 6 mice. The BU group has 7 mice. ** *p* < 0.01 versus NC group; # *p* < 0.05 versus MD group; ## *p* < 0.01 versus MD group.

Previous clinical studies have demonstrated that the intestinal abundance of *B. uniformis* is significantly higher in healthy controls than that in IBD patients [7,8], suggesting a potential role of *B. uniformis* in protecting against the development of UC in human gut. In this regard, we next investigated the effect of *B. uniformis* F18-22 on DSS-induced UC in mice. Interestingly, we found that oral intake of *B. uniformis* F18-22 for seven days significantly retarded the body weight loss, alleviated colon contraction, reduced incidences of intestinal bleeding, and improved stool consistency in diseased mice (Figure 3A–D). Taken together, these results provide the first evidence for the anti-colitis effect of *B. uniformis* F18-22 in DSS-fed mice. 

**Figure 4:**
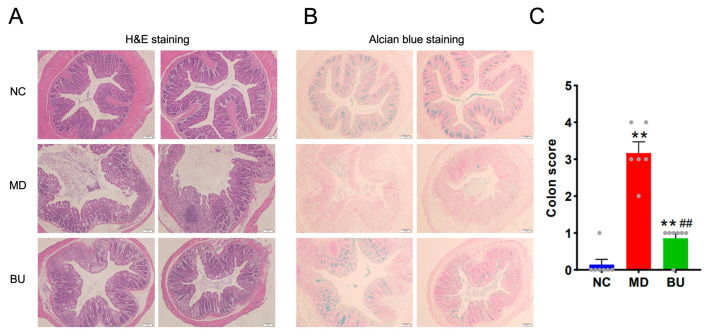
Oral administration of *B. uniformis* F18-22 attenuated DSS-induced mucosal damage. H&E staining of the colonic tissues of the mice (**A**). Alcian blue staining of the colonic tissues of the mice (**B**). Colon score analysis based on H&E staining (**C**). Each gray dot in the panel C represents one mouse in the experiments. The NC group has 7 mice. The MD group has 6 mice. The BU group has 7 mice. ** *p* < 0.01 versus NC group; ## *p* < 0.01 versus MD group.

Accumulated evidence has indicated that DSS-induced UC is associated with intestinal mucosal damage in the gut [14]. Therefore, we next performed H&E staining and Alcian blue staining to further explore the anti-colitis effect of *B. uniformis* F18-22. As expected, oral intake of *B. uniformis* F18-22 successfully protected the mice from DSS-induced disruption of the colonic epithelial layer (Figure 4A–C). Altogether, dietary intake of *B. uniformis* F18-22 improved UC and ameliorated intestinal mucosal damage in DSS-fed mice (Figure 3 and Figure 4). Collectively, these results provide new insights into the role of *B. uniformis* in the development of UC in the human gut. 

### 2.3. B. uniformis F18-22 Improved Gut Dysbiosis by Increasing the Abundance of Eubacterium siraeum and Decreasing the Amount of Escherichia-Shigella spp. in DSS-Fed Mice

**Figure 5:**
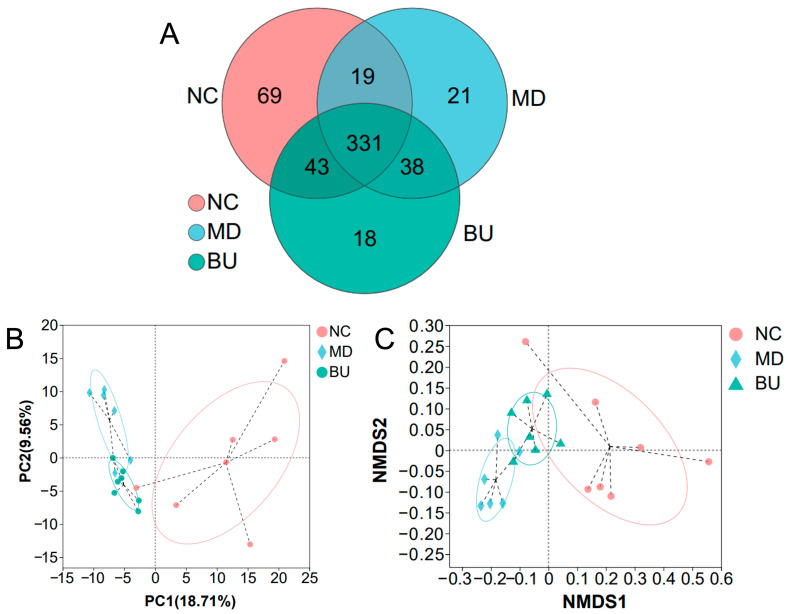
Oral administration of *B. uniformis* F18-22 significantly changed the structure of the gut microbiota in DSS-fed mice. Venn diagram analysis of the composition of gut microbiota at the operational taxonomic units (OTUs) level (**A**). PCA score plot analysis of the gut microbiota (**B**). NMDS score plot analysis of the gut microbiota (**C**).

Previous studies have demonstrated that gut microbiota plays a pivotal role in the pathogenesis of UC [4,5,6]. In this sense, we next investigated the effect of *B. uniformis* F18-22 on the composition of the gut microbiota using 16S high-throughput sequencing. Interestingly, we found that oral administration of *B. uniformis* F18-22 significantly changed the structure of the gut microbiota in diseased mice (Figure 5A). Specifically, as indicated via PCA and NMDS analyses, *B. uniformis* F18-22 treatment induced a remarkable shift of the gut microbiota structure in diseased mice towards that in NC mice (Figure 5B,C). 

**Figure 6:**
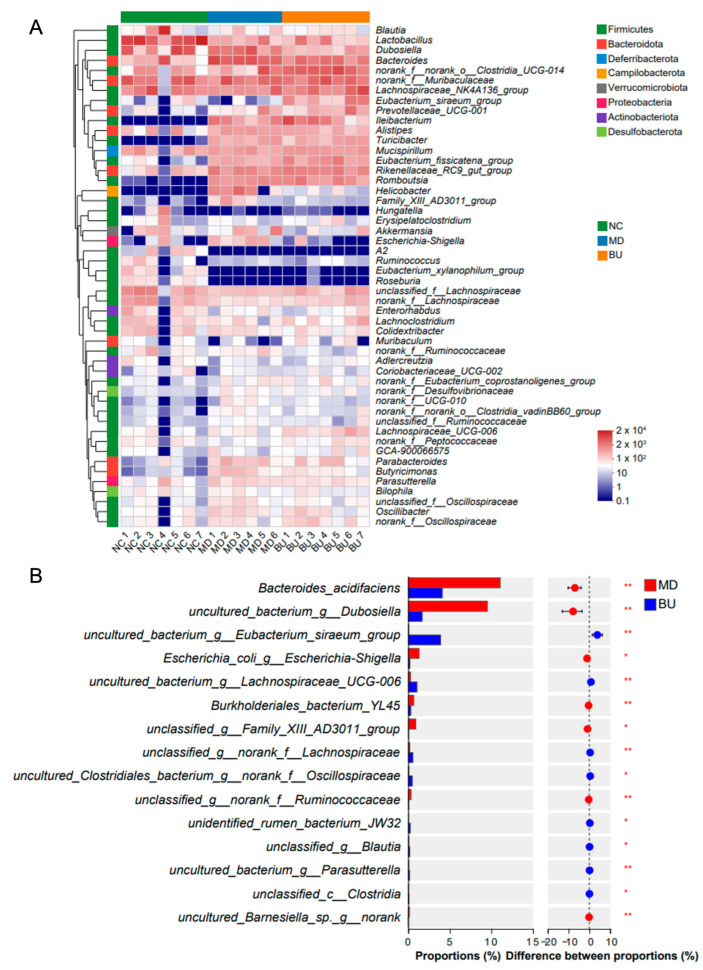
Oral administration of *B. uniformis* F18-22 improved gut dysbiosis in DSS-fed mice. Heatmap analysis of the composition of gut microbiota (**A**). Wilcoxon rank-sum test analysis (**B**). * *p* < 0.05; ** *p* < 0.01.

Further analysis indicated that dietary intake of *B. uniformis* F18-22 modulated the composition of the gut microbiota in DSS-fed mice at both phylum and genus levels (Figure 6A and Figure S1). A Wilcoxon rank-sum test was performed to compare the structural differences of the gut microbiota at the species level between two groups (Figure 6B and Figure S2). Interestingly, *B. uniformis* F18-22 improved gut dysbiosis in DSS-fed mice by increasing the abundance of anti-inflammatory acetate-producing bacterium *Eubacterium siraeum* and decreasing the amounts of pro-inflammatory pathogenetic bacteria, *Escherichia-Shigella* spp., and *Bacteroides acidifaciens* (Figure 6B). 

### 2.4. B. uniformis F18-22 Was Well Tolerated in Mice and Showed No Oral Toxicity after Repeated Administration

**Figure 7:**
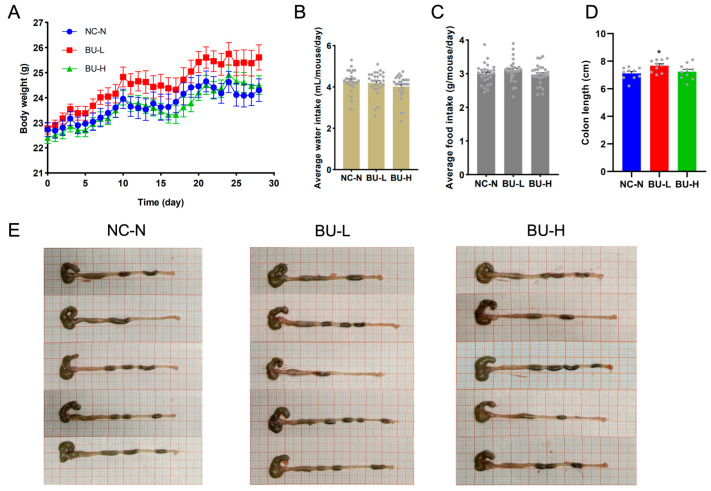
*B. uniformis* F18-22 was well tolerated in mice. Body weight change of the mice (**A**). Average water intake of the mice (**B**). Average food intake of the mice (**C**). Each gray dot in the panel (**B**,**C**) represents the value of the daily water intake and daily food intake. The water intake and food intake were monitored for 28 days. Colon length (**D**). Each gray dot in the panel (**D**) represents one mouse in the experiments. The NC-N group has 10 mice. The BU-L group has 10 mice. The BU-H group has 10 mice. Representative morphology of the colon (**E**). * *p* < 0.05 versus NC-N group.

We next investigated the safety profiles of *B. uniformis* F18-22 in healthy mice. Notably, we found that oral administration of *B. uniformis* F18-22, both at a low and at a high dosage, exerted no effect on body weight, water and food intake of the mice (Figure 7A–C). Intriguingly, low-dosage treatment of *B. uniformis* F18-22 slightly increased the colon length whereas high-dosage treatment of *B. uniformis* F18-22 had no such effect (Figure 7D). However, both low-dosage treatment and high-dosage treatment of *B. uniformis* F18-22 did not have any effects on stool consistency and fecal pellet morphology (Figure 7E). Collectively, these results indicate that *B. uniformis* F18-22 is well tolerated in mice even dosed at 10 times the therapeutic concentration. 

**Figure 8:**
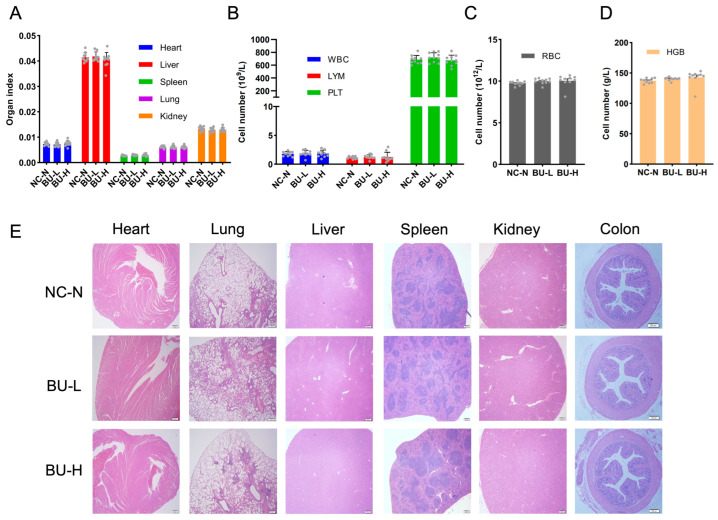
*B. uniformis* F18-22 showed no toxic effect on the major organs of mice. Organ index (**A**). Numbers of the white blood cells, lymphocytes, and platelets (**B**). Numbers of the red blood cell (**C**). Hemoglobin concentrations in the blood of the mice (**D**). Each gray dot in the panel (**A**–**D**) represents one mouse in the experiments. The NC-N group has 10 mice. The BU-L group has 10 mice. The BU-H group has 10 mice. H&E staining of the heart, lung, liver, spleen, kidney, and colon (**E**).

Further analysis indicated that *B. uniformis* F18-22 had no toxic effect on the major organs of mice including heart, liver, spleen, lung, kidney, and colon (Figure 8A,E). Additionally, both low-dosage treatment and high-dosage treatment of *B. uniformis* F18-22 treatment did not change the numbers of white blood cells (WBCs), lymphocytes (LYMs), platelets (PLTs) and red blood cells (RBCs) of the mice (Figure 8B,C). Moreover, the hemoglobin (HGB) concentrations in the blood of the mice were also not affected by *B. uniformis* F18-22 treatment (Figure 8D). Taken together, *B. uniformis* F18-22 was well tolerated in mice and showed no oral toxicity even after repeated daily administration for 28 consecutive days.

## 3. Discussion

### 3.1. Potential Applications of B. uniformis F18-22

**Figure 9:**
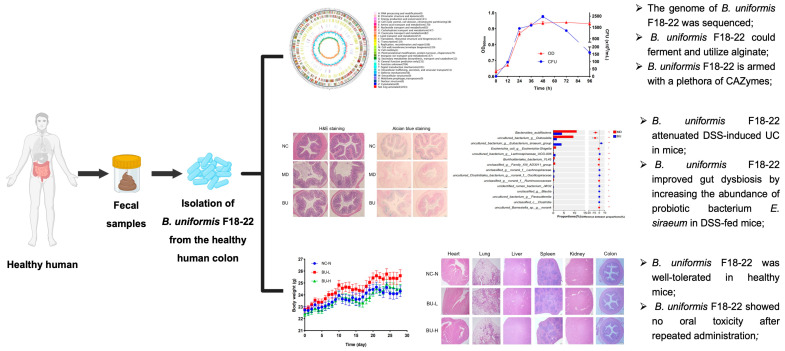
Summary of the main findings of the present study. Part of the figure was created with BioRender.com (accessed on 13 September 2023). * *p* < 0.05; ** *p* < 0.01.

Previous clinical studies have demonstrated that the intestinal abundance of *B. uniformis* is significantly higher in healthy controls than that in UC patients [7,8]. However, what effect *B. uniformis* has on the development and pathogenesis of UC has not been characterized. Here, we show for the first time that *B. uniformis* F18-22, an alginate-fermenting bacterium with good safety profiles from the healthy human colon, protects against DSS-induced UC in mice. Our study paves the way for the research and development of *B. uniformis* F18-22 as a next-generation probiotic bacterium (Figure 9).

### 3.2. Limitations of the Current Study

Our study has some limitations. First, although we have sequenced and analyzed the whole genome of *B. uniformis* F18-22, we are still at the very beginning of understanding the metabolic characteristics of this new probiotic bacterium. Many COGs in the genome have not been successfully annotated (Figure 1). Second, *B. uniformis* F18-22 is armed with a plethora of CAZymes in its genome (Figure 2). It is possible that *B. uniformis* F18-22 could degrade and ferment a wide range of complex carbohydrates in our daily diet. However, we have only tested alginate in the present study and we do not know if it is the best carbon source for *B. uniformis* F18-22. Third, based on our results, we could not determine whether or not *B. uniformis* F18-22 has successfully colonized the gastrointestinal tract in DSS-fed mice. The metabolic fate of *B. uniformis* F18-22 after oral administration has not been explored.

### 3.3. Future Directions for the Study of B. uniformis F18-22

*Escherichia-Shigella* spp. and *B. acidifaciens* are pathogenic colitis-associated bacteria in the gut [14,15]. In contrast, *E. siraeum* is an anti-inflammatory acetate-producing probiotic bacterium in the colon [16,17]. Our results suggest that *B. uniformis* F18-22 could attenuate gut dysbiosis in DSS-fed mice by promoting the growth of *E. siraeum* and inhibiting the proliferations of *Escherichia-Shigella* spp., and *B. acidifaciens*. The present study provides the first evidence for understanding the therapeutic effects of *B. uniformis* F18-22 on UC from the perspective of dysbiotic gut microbiota. However, more detailed investigations are still needed to fully characterize the anti-colitis mechanisms of this probiotic bacterium.

*B. uniformis* CECT 7771 is a symbiont bacterium from the colon of healthy infants [10,18]. Previous studies have indicated that oral consumption of *B. uniformis* CECT 7771 for 90 days is safe in experimental rats [18]. Similarly, we found that *B. uniformis* F18-22 was well tolerated in mice and showed no oral toxicity after repeated administration for 28 consecutive days even dosed at 10 times the therapeutic concentration. Altogether, these results suggest that the probiotic strains of *B. uniformis* from healthy individuals generally have a good safety profile in rodents. More studies are therefore warranted to further assess the safety of *B. uniformis* F18-22 in humans.

Our study indicates that the genome of *B. uniformis* F18-22 encodes many CAZymes, including glycoside hydrolases (GHs), glycosyltransferases (GTs), polysaccharide lyases (PLs), carbohydrate esterases (CEs), carbohydrate-binding modules (CBMs) and auxiliary activities (AAs). CAZymes are the most important enzymes for the metabolism of complex carbohydrates in the human diet [19,20,21,22]. These results indicate that *B. uniformis* F18-22 might be able to ferment and utilize a wide range of dietary polysaccharides in the gut. This could possibly give *B. uniformis* F18-22 a competitive advantage to survive in the colon when the carbon source is changed between different diets. Nonetheless, more intensive examinations are needed to verify this possibility. 

## 4. Materials and Methods

### 4.1. Chemicals and Reagents

The standard short-chain fatty acids (SCFAs) solutions, including lactate, acetate, propionate, succinate, and butyrate were all purchased from Sigma-Aldrich (St. Louis, MO, USA). Tryptone, peptone, yeast extract, and Tween 80 used for the in vitro anaerobic fermentation experiments were all obtained from Sigma-Aldrich (St. Louis, MO, USA). Dextran sulfate sodium (DSS) was purchased from MP Biomedicals (Solon, OH, USA). DSS was used to induce ulcerative colitis in the colon of C57BL/6J mice. 

Hemin, alginate, agar, and L-cysteine hydrochloride were acquired from Sangon Biotech (Shanghai, China). These chemicals were added to the VI medium and were used for the anaerobic culture of the bacterium *B. uniformis* F18-22. All other chemicals of analytical grade used in the present study were purchased from Sinopharm Chemical (Shanghai, China) unless otherwise specified.

### 4.2. In Vitro Fermentation

*B. uniformis* F18-22 is an alginate-fermenting bacterium that has been previously isolated from the healthy human colon [12]. Therefore, the VI culture medium containing alginate as a major carbon source at 8 g/L was applied to investigate the fermentation characteristics of *B. uniformis* F18-22 as previously described [12,13]. The fermentation experiments were carried out anaerobically (80% N_2_, 10% H_2_ and 10% CO_2_) at 37 °C in an Electrotek AW 500SG anaerobic chamber (Shipley, West Yorkshire, UK). 

The SCFAs produced during the in vitro anaerobic fermentation were analyzed and monitored using the well-established high-performance liquid chromatograph (HPLC) (Agilent 1260, Santa Clara, CA, USA) method as previously described [12,13]. Transmission electron microscope (TEM) analysis of the bacterial cell morphology of *B. uniformis* F18-22 was conducted by Servicebio Technology (Wuhan, China) using the HT7800/HT7700 microscope from Hitachi High-Tech (Shanghai, China). The bacteria used for the TEM analysis were collected at the exponential phase of growth at about 20 h (Figure 2B). 

### 4.3. Animals and Treatment

All the specific-pathogen-free (SPF) animals used in the present study were obtained from Beijing Vital River Laboratory Animal Technology (Beijing, China) (Certificate No. SCXK (Jing) 2016-0011). The animal experiments were approved by the Ethical Committee of Ocean University of China, School of Medicine and Pharmacy (Permission No. OUC-2022-0901-01). The experiments in the present study were performed in good compliance with the Guide for the Care and Use of Laboratory Animals (National Academies Press, 8th edition, 2011) [23]. 

In the first animal experiment, a total of 20 male 8-week-old C57BL/6J SPF mice were utilized to investigate the therapeutic effects of *B. uniformis* F18-22 on DSS-induced UC. The mice were randomly divided into 3 different treatment groups: the normal control group (NC, *n* = 7), the model group (MD, *n* = 6), and the *B. uniformis* F18-22 treatment group (BU, *n* = 7). *B. uniformis* F18-22 was given daily at a dosage of about 1.00 × 10^8^ colony-forming units (CFUs)/day/mouse via gavage for 7 consecutive days. *B. uniformis* F18-22 was cultured in the VI culture medium containing alginate as a major carbon source at 8g/L. The bacteria were collected at the exponential phase of growth at about 20 h. 

Mice in the MD and BU groups were given free access to 2.0% (*w*/*v*) DSS in the daily drinking water for 6 consecutive days. All mice were humanely sacrificed on the 7th day. The colon tissues of the mice were harvested for hematoxylin and eosin (H&E) staining and Alcian blue staining. The cecum tissues were collected for gut microbiota analysis. The symptom scores were calculated based on the stool morphology, intestinal bleeding occurrence and body weight change of the mice at the last day of the experiment. The histopathological colon scores were determined based on H&E staining as previously described [14].

In the second animal experiment, a total of 30 male 8-week-old C57BL/6J SPF mice were applied to further explore the safety profile of *B. uniformis* F18-22. All mice were randomized into 3 different treatment groups: the normal control group (NC-N, *n* = 10), the low-dosage treatment group (BU-L, *n* = 10), and the high-dosage treatment group (BU-H, *n* = 10). In the BU-L group, *B. uniformis* F18-22 was given daily at a dosage of about 1.00 × 10^8^ CFUs/day/mouse by gavage for 28 consecutive days, whereas in the BU-H group, *B. uniformis* F18-22 was given daily at a dosage of about 1.00 × 10^9^ CFUs/day/mouse by gavage for 28 consecutive days. *B. uniformis* F18-22 was cultured in the VI culture medium containing alginate as a major carbon source at 8 g/L. The bacteria were collected at the exponential phase of growth at about 20 h. 

The average water intake and food intake of the mice were monitored every day. All mice were humanely sacrificed on the last day of the experiment. The blood cells of the mice were analyzed using an automatic blood cell analyzer (RT-7600Vet) from Rayto Life and Analytical Sciences (Shenzhen, China). The major organs of the mice were collected and the organ index was calculated and compared between different treatment groups. The H&E staining of the heart, lung, liver, spleen, kidney, and colon was performed as previously described [12,14]. 

### 4.4. High-Throughput Sequencing and Bioinformatic Analyses

*B. uniformis* F18-22 was cultured in the VI culture medium containing alginate as a major carbon source at 8 g/L. The bacteria were collected at the exponential phase of growth at about 20 h. The genomic DNA of *B. uniformis* F18-22 was extracted and the complete genome was sequenced using the Oxford Nanopore Technologies (ONT) Nanopore PromethION platform from Biomarker Technologies (Beijing, China). The genome analysis was conducted using the online tools from BMKCloud (www.biocloud.net (accessed on 1 August 2023)). Clusters of Orthologous Groups (COGs) function analysis and carbohydrate-active enzymes (CAZymes) analysis of the genome were conducted to explore the metabolic potential of *B. uniformis* F18-22.

Phylogenetic tree analysis of *B. uniformis* F18-22 was performed based on the 16S sequences of the bacteria using the Molecular Evolutionary Genetics Analysis (MEGA) software (version 7.0.26) [12,24]. The metagenomic DNAs of the mice’s cecal microbiota were extracted using a Qiagen QIAamp DNA Stool Mini Kit (Hilden, Germany). The 16S V3-V4 hypervariable gene regions were specifically amplified using the well-established universal primers, 338F (ACTCCTACGGGAGGAGCAG) and 806R (GGACTACHVGGGTWTCTAAT), as previously described [12,13,14]. The obtained amplicons were sequenced and analyzed using an Illumina PE300 platform from Majorbio Bio-pharm Biotechnology (Shanghai, China). Bioinformatic analyses of the sequencing data, including Wilcoxon rank-sum test, Venn diagram, non-metric multidimensional scaling (NMDS) score plot, principal components analysis (PCA) and heatmap analysis were all conducted using the online tools from Majorbio Cloud Platform (www.majorbio.com (accessed on 1 August 2023)) [25]. 

### 4.5. Statistical Analyses

All results were expressed as the mean ± standard error of mean (SEM). The statistical analyses were performed using Student’s t-test from the GraphPad Prism 8.0.2 software (Boston, MA, USA). The Wilcoxon rank-sum test was performed to compare the structural differences of the gut microbiota between two groups. The results were considered statistically significant at *p* < 0.05. * *p* < 0.05; ** *p* < 0.01.

## 5. Conclusions

Dietary intake of *B. uniformis* F18-22, an alginate-fermenting bacterium from the healthy human colon, protects against DSS-induced UC in mice. Specifically, *B. uniformis* F18-22 retarded body weight loss, alleviated colon contraction, reduced incidences of intestinal bleeding, and improved stool consistency in diseased mice. Additionally, *B. uniformis* F18-22 improved gut dysbiosis in UC mice by increasing the abundance of anti-inflammatory acetate-producing bacterium, *Eubacterium siraeum*, and decreasing the amount of pro-inflammatory pathogenetic bacteria, *Escherichia-Shigella* spp. and *B. acidifaciens*. Moreover, *B. uniformis* F18-22 was well tolerated in mice and showed no oral toxicity after repeated daily administration for 28 consecutive days. Taken together, our study illustrates for the first time that *B. uniformis* F18-22 is a safe and novel probiotic bacterium for the treatment of UC from the healthy human colon.

## Data Availability

The data presented in this study are available on request from the corresponding authors.

## Supplementary Materials

The following supporting information can be downloaded at https://www.mdpi.com/article/10.3390/ijms241914669/s1.
