# Supplementary material for: Discovery of Bacteroides uniformis F18-22 as a Safe and Novel Probiotic Bacterium for the Treatment of Ulcerative Colitis from the Healthy Human Colon
Source: Int J Mol Sci. 2023 Sep 28;24(19):14669. doi: 10.3390/ijms241914669 (PMC10572632; doi:10.3390/ijms241914669)
Supplement: Supplementary file 1 [file ijms-24-14669-s001.zip › ijms-2608780-supplementary.pdf]

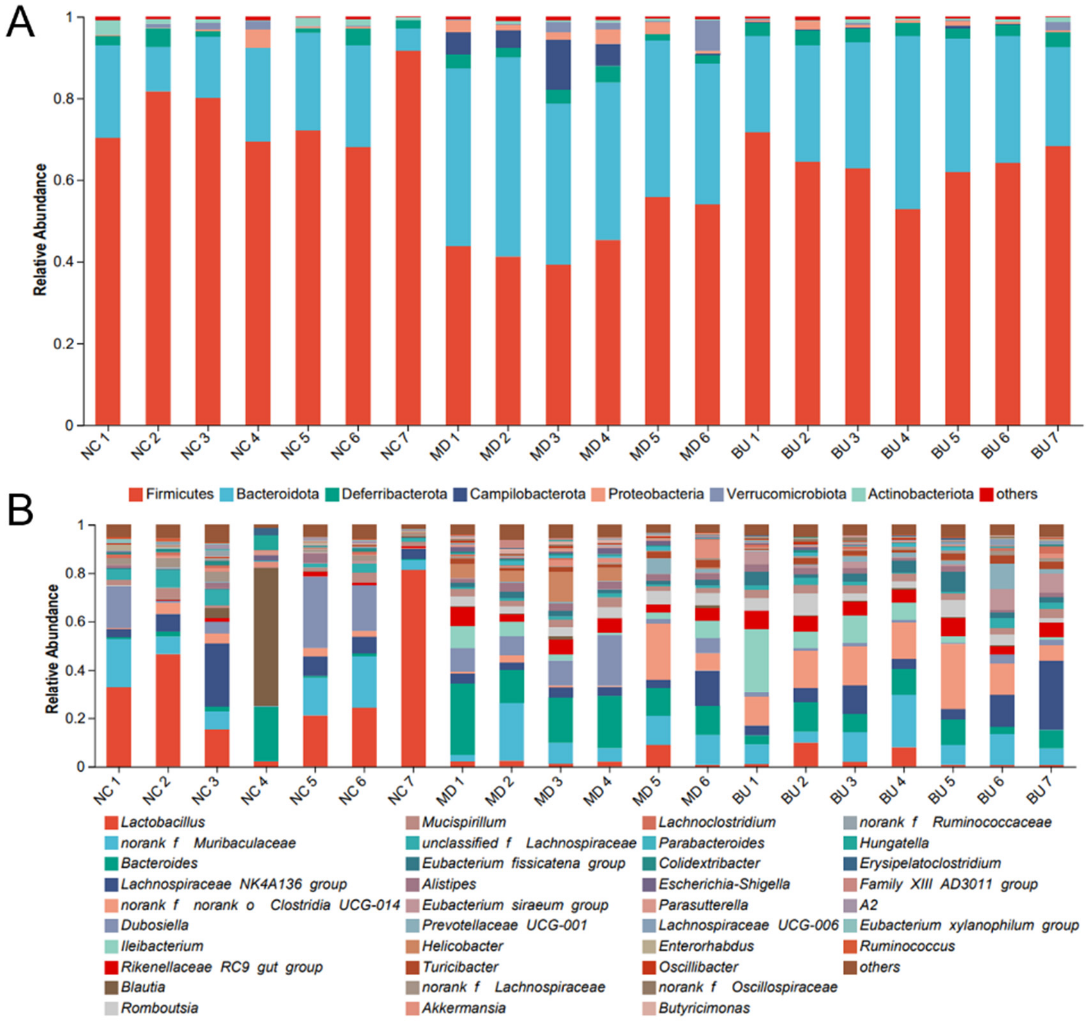

Figure S1. Composition of the gut microbiota at the phylum level (A) and the genus level (B).

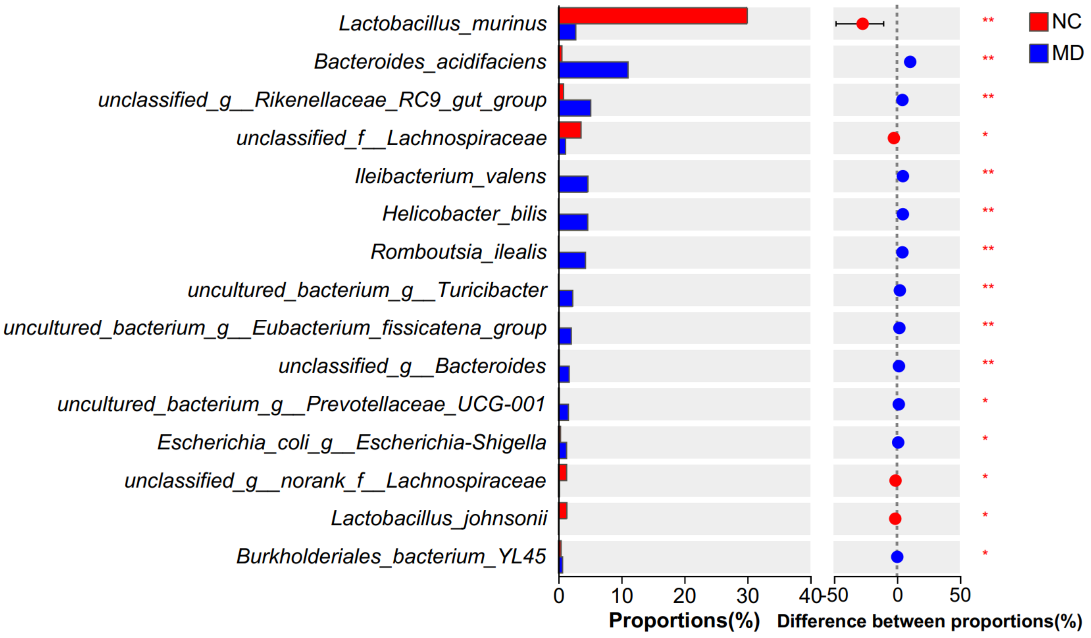

Figure S2. Wilcoxon rank-sum test analysis of the gut microbiota at the species level.
